# Optical coherence tomography angiography of the foveal avascular zone in diabetic retinopathy

**DOI:** 10.1007/s00417-015-3148-2

**Published:** 2015-09-04

**Authors:** Florentina J. Freiberg, Maximilian Pfau, Juliana Wons, Magdalena A. Wirth, Matthias D. Becker, Stephan Michels

**Affiliations:** Department of Ophthalmology, Stadtspital Triemli, Birmensdorferstrasse 497, CH-8063 Zurich, Switzerland; University of Zurich, Zurich, Switzerland; University of Heidelberg, Heidelberg, Germany

**Keywords:** Optical coherence tomography angiography, Foveal avascular zone, Diabetic retinopathy

## Abstract

**Purpose:**

To analyze foveal avascular zone (FAZ) dimensions and symmetry in patients with diabetic retinopathy (DR) compared to healthy controls using optical coherence tomography angiography (OCT angiography).

**Methods:**

OCT angiography was performed via an Avanti® RTVue 100 XR OCT system (Optovue, Inc., Fremont, CA, USA) in patients with diabetes mellitus (DM) and healthy adults. A frame centered on the fovea was used for FAZ measurements. The borders of the superficial vascular layer were defined as 3 μm below the internal limiting membrane (ILM) and 15 μm below the inner plexiform layer (IPL), and for the deep vascular layer as15 μm and 70 μm below the IPL, respectively. Angles of maximum FAZ diameter were measured in all eyes by two graders.

**Results:**

In healthy eyes (*N* = 25), the FAZ surrounding vascular arcades were intact, showing a vertical or horizontal oval symmetrical formation with a maximum diameter usually on the horizontal or vertical axis. Diabetic eyes (*N* = 29) presented with disintegrity of the vascular arcades, resulting in an enlarged FAZ. In the superficial layer, the mean horizontal FAZ diameter was significantly larger in the DR group (753 μm ±272 μm) than in the control group (573 μm ±177 μm, *p* = 0.029). The difference was even more pronounced in the deep layer, with a mean value of 659 μm ±194 μm in the control group and 1009 μm ±342 μm in the DR group (*p* = 0.001). Furthermore, in the superficial layer, the angle of the maximum FAZ diameter was 0° (±15°) or 90° (±15°) in 72.0 % of healthy eyes. In eyes with DR, the angle was 0° (±15°) or 90° (±15°) in only 6.9 % of cases, due to the irregular configuration of the FAZ.

**Conclusions:**

OCT angiography is capable of imaging retinal vasculature without dye injection. Our data suggest that it can detect disintegrity of the vascular arcades surrounding the FAZ, thus differentiating DM from healthy eyes. Vascular abnormalities were more pronounced in the deep vascular layer.

## Introduction

Diabetic retinopathy (DR) is the leading cause of blindness and visual impairment in adults of working age in developed countries, with an increase in affected individuals predicted [1].

For DR, multiple funduscopic abnormalities of the retinal vasculature and foveal avascular zone (FAZ) have been described [2]. Studies have found that the maximum and mean diameter and the FAZ circumference are significantly greater in diabetic eyes compared to healthy eyes [3, 4]. Similar correlations have been reported for FAZ measurements based on fluorescein angiography (FA) [5]. The underlying pathophysiology of FAZ enlargement in DR is most likely related to microinfarction within the surrounding vascular arcades [3–6]. With the 1991 revision of the Early Treatment Diabetic Retinopathy Study (ETDRS) grading criteria for DR, which emerged from the funduscopic Airlie House classification, criteria were extended to include FA [2, 7]. Among the FA-based grading criteria were arteriolar abnormalities, abnormalities of the retinal pigment epithelium, cystoid changes, capillary loss (ischemia) and leakage of fluorescein dye [7].

Today, FA and optical coherence tomography (OCT) are commonly used imaging modalities in DR. The first report describing FA to visualize the retinal vasculature was published in 1930 by Kikai [8]. The method was refined through the addition of a motion picture camera and an exciter filter by Flocks et al. in 1959 [9]. Shortly thereafter, MacLean and Maumenee published the first case of the use of FA to obtain a diagnosis [10]. The first protocol of standardized serial FA photography, which eventually made its way into clinical practice, was presented by Novotny and Alvis in 1961 [11]. However, the disadvantages of FA soon became apparent. Studies showed that FA photographs correlated only with the anatomical arrangement of large superficial retinal vessels located within the nerve fiber layer and the ganglion cell layer, and perfusion of deeper retinal vasculature was not visible in FA photographs [12, 13]. Light scattering within the retina has been suggested as the underlying cause of the non-visibility of small retinal vessels in the ganglion cell layer and inner nuclear layer [14]. As it is an invasive imaging technique, FA has been associated with rare but life-threatening complications such as anaphylaxis and cardiac arrest [15].

Optical coherence tomography (OCT), which was introduced to the scientific community in 1991, revolutionized the clinical examination of the anterior and posterior segment of the eye [16, 17]. OCT angiography, which has recently been developed, is a technique based on de-correlation between the signals of two sequential OCT cross-sectional scans repeated at the same location caused by blood flow [18–25]. Spaide et al. demonstrated in 2015 that OCT angiography could image all layers of the retinal vasculature, including the deep layers, in contrast to FA imaging [26, 27].

With regard to the age of affected individuals and the predicted increase of DR in the population, early detection plays a pivotal role in the treatment of DR. With the imaging modalities currently used, the deep vasculature cannot be made visible. Thus it is of great interest to determine whether OCT angiography is able to detect differences in the superficial and deep retinal vasculature and differences in the FAZ, and therefore might be helpful for the early recognition and grading of DR.

This retrospective single-center study was designed to investigate the dimensions and shape of the FAZ in patients with DR in comparison to a healthy control group, using OCT angiography. An additional objective was to investigate differences in FAZ configuration between the superficial and deep vascular layers.

## Material and methods

Adults with healthy eyes and patients with DR were evaluated with OCT angiography. Healthy eyes and patients with DR were selected and age-matched. DR was diagnosed in all patients by indirect slit-lamp funduscopy with pupil-dilatation, digital color fundus photography (CFP) and FA and graded according to the ETDRS classification.

In accordance with the tenets of the Declaration of Helsinki, the study was reviewed and approved by the local ethics committee in Zurich. Written informed consent was collected from all study subjects prior to investigation-related procedures.

All patients were imaged at the department of ophthalmology of the Triemli Hospital in Zurich, Switzerland. This study evaluated patients diagnosed with and treated for DR and healthy adults scheduled for regular visits.

OCT angiography was performed with an Avanti RTVue XR system (Optovue, Inc., Fremont, CA, USA). The instrument has an A-scan rate of 70,000 scans per second, using a scan beam centered at 840 nm, with a bandwidth of 45 nm. Each OCT angiography volume contained 304 × 304 A-scans, with two consecutive B-scans (M-B frames) captured at each fixed position before proceeding to the next sampling location. The acquisition time per volume amounted to approximately 3 s. Two orthogonal volumes were acquired in order to perform motion correction and to minimize motion artifacts. The OCT angiography images are based on a split-spectrum amplitude de-correlation algorithm (SSADA). The images are average de-correlation values viewed perpendicularly through the thickness (slabs) being evaluated. The images were captured with the standard macula protocol with a resolution of 2 mm × 2 mm. For all measurements, the automated segmentation with the preset settings for the superficial vascular layer and deep vascular layer was utilized. Hereby, the upper border of the superficial vascular layer was defined as 3 μm below the internal limiting membrane (ILM) and the lower border as 15 μm below the inner plexiform layer (IPL). For the deep vascular layer, the borders were defined as 15 μm and 70 μm below the IPL, respectively.

Additionally, standard spectral-domain OCT (SD-OCT) scans (512 A-scans, 20° × 15°) were taken of all eyes using the Heidelberg Spectralis^®^ system (Heidelberg Engineering, Heidelberg, Germany). During the visit, the current best-corrected visual acuity (BCVA) and the intraocular pressure (IOP) were measured. Furthermore, medical charts of all patients were reviewed for basic demographics, clinical data and previous FA images

Image processing and analysis using ImageJ software (National Institutes of Health [NIH], Bethesda, MD, USA) was performed independently by two graders (F.F., M.P.) who were each masked to the results of the other. The use of the thresholding function of ImageJ to create binary images for better contrast and the identification of erroneous auto-segmentation planes was left to the discretion of the graders. The integrated ImageJ line tool was used to measure the diameters and angles. All results were converted to micrometers by multiplying the measured values by 6.579 μm/pixel. The intraclass correlation coefficient (ICC) of the graders amounted to 0.857 (ICC 2.1) for the measurement of diameters. For statistical purposes, Snellen visual acuity fractions were transferred to ETDRS letter scores according to Gregori et al. [28] All statistical analyses were performed using SPSS^®^ version 20 statistical software (IBM Corp., Armonk, NY, USA). Since distributions were non-normal according to the Shapiro–Wilk test, non-parametric tests were performed for all analyses. The Mann–Whitney *U* test was used to test between two independent groups when the dependent variable was either ordinal or continuous. For statistical testing of the categorical data, Pearson's chi-square test (χ^2^) was used. Lastly, as a non-parametric measure of statistical dependence between two variables, Spearman's rho was applied.

## Results

### Clinical demographics

The study was carried out on a total of 54 eyes of 37 patients. DR was present in 29 eyes of 15 patients. A total of 25 healthy eyes in 22 patients were matched according to age (Table 1).

**Table 1:** Clinical demographics

Patient Characteristics (*N* = 54 )		
	Control Group	Diabetic Retinopathy Group
Age, years (mean ± SD)	60.41 ± 20.35	58.58 ± 13.22
Gender, male/female, *N*	15/10	24/5
Mean BCVA, ETDRS	83.64 ± 3.6	74.7 ± 15.5
Diabetic retinopathy, *N*	0	29
Proliferative diabetic retinopathy	0	13
Pre-proliferative diabetic retinopathy	0	4
Non-proliferative diabetic retinopathy	0	18
Mild	0	6
Moderate	0	5
Severe	0	7
Diabetic macular edema	0	22
Lens status, *N*		
Phakic	19	22
Pseudophakic	6	7
Laser photocoagulation, *N*		
Panretinal	0	15
Focal	0	11

*N* number, *SD* standard deviation, *BCVA* best-corrected visual acuity, *ETDRS* Early Treatment Diabetic Retinopathy Study

The mean age was 58.58 years (standard deviation [SD] ±13.22 years) in the DR group and 60.41 years (±20.35) in the control group. No statistically significant difference in age was found between groups (*p* = 0.41).

### Qualitative findings

**Fig. 1:**
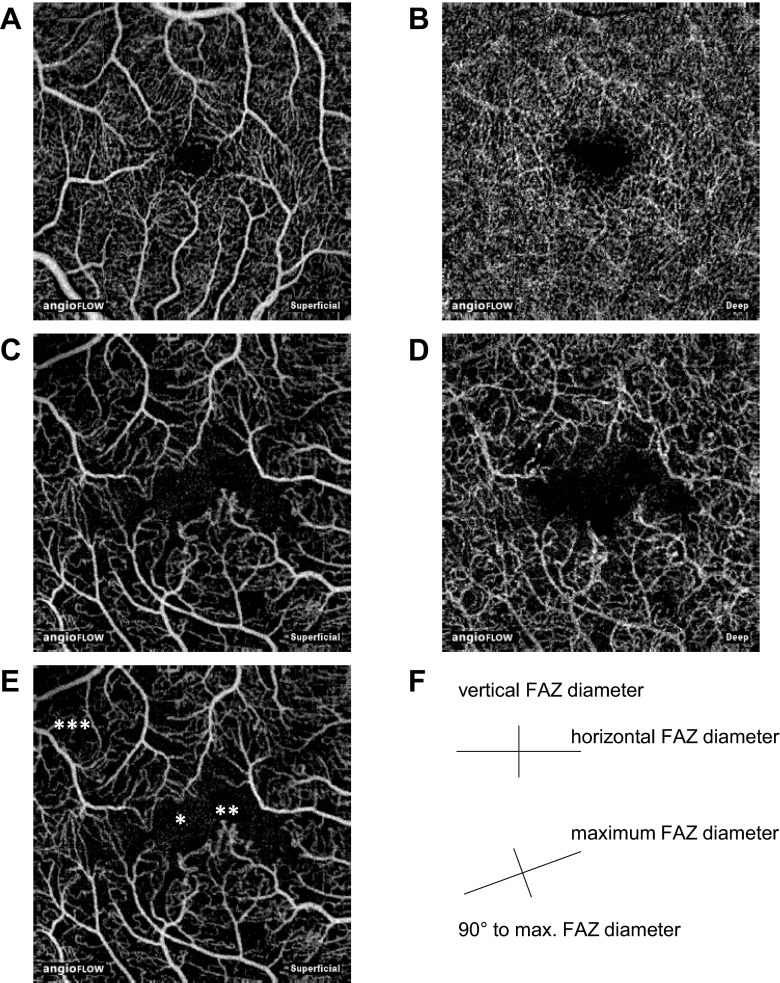
The superficial and deep FAZ of a healthy eye (**a**, **b**) and an eye with moderate non-proliferative diabetic retinopathy (**c**, **d**). Disintegrity of the vascular arcades with subsequent enlargement of the FAZ (*), microaneurysms and vascular abnormalities (**) and of reduced capillary density (***) can be identified with OCT angiography in the eye with DR (**e**). The size of all images is 3 mm × 3 mm. The schematic diagram exemplifies the different diameters, which were measured for this study(**f**)

In the control group, the FAZ surrounding vascular arcades, a circular system of capillaries, was intact in all 29 eyes, showing either a vertical or horizontal oval-shaped symmetrical formation without gaps, holes or interruption of the capillary network. The maximum diameter of the FAZ in healthy eyes with a horizontal oval-shaped FAZ was on the horizontal axis in the majority of cases. Analogously, in eyes with a vertical oval-shaped FAZ, the maximum diameter was typically on the vertical axis (Fig. 1).

**Fig. 2:**
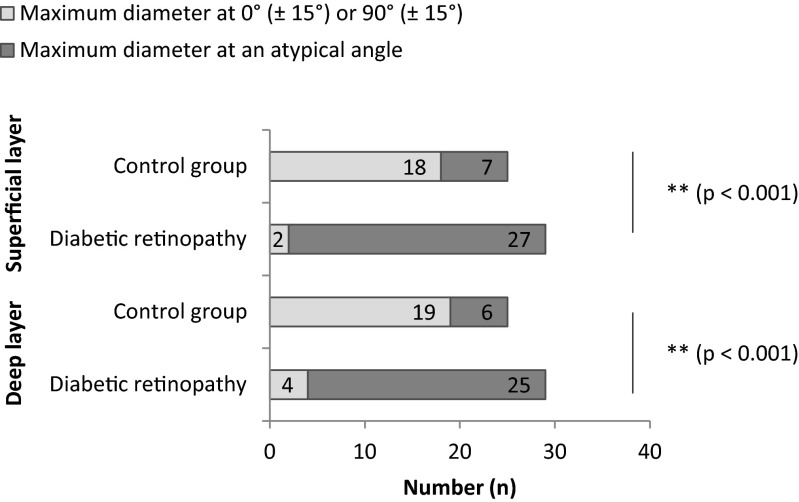
An angle of the maximum FAZ diameter of 0° (±15°) or 90° (±15°) was evaluated as normal. All other angles were defined as atypical. Pearson's chi-squared test (χ2) was used for statistical testing of the categorical data

In contrast, the DR group presented the classical features of diabetic microangiopathy. Areas of capillary non-perfusion (depicted on previous FA) appeared as areas of reduced capillary density in OCT angiography, with a predominance in the deep layer. Besides rarefaction of capillaries, venous beading and loops, intraretinal microvascular abnormalities and microaneurysms were visible in OCT angiography. Since OCT angiography does not distinguish flow directionality, CFP was used for veins. Interestingly, diabetic eyes often presented with a disintegrity of the vascular arcades surrounding the FAZ, resulting in an enlargement of the FAZ. The FAZ often appeared to be asymmetrical due to gaps, holes or interruption of the capillary network. Consequently, in the DR group the angle of the maximum diameter of the FAZ was usually neither on the horizontal nor on the vertical axis (see Fig. 2).

Other clinical findings, such as retinal hemorrhages and cystoid macular edema, reduced the de-correlation signal. Thus, these findings caused shadowing, if present, in proximity to a vascular structure.

### The angle of the maximum diameter of the FAZ can be used to differentiate healthy eyes from eyes with diabetic retinopathy

As mentioned previously, in healthy eyes the maximum FAZ diameter was usually either horizontal (oval shaped FAZ) or vertical (circular shaped FAZ). In contrast, in eyes with DR atypical angles of the maximum FAZ diameter were common due to the disintegrity of the surrounding vascular arcades.

As shown in Fig. 2, in healthy eyes the angle of the maximum FAZ diameter was either 0° (±15°) or 90° (±15°) in 72.0 % of all cases in the superficial layer. In the deep layer a typical (either 0° [±15°] or 90° [±15°]) angle was found in 76.0 % of all healthy eyes.

On the contrary, in eyes with DR, the angle of the maximum FAZ diameter amounted only in 6.9 % of all cases either to 0° (±15°) or 90° (±15°) in the superficial layer. In the deep layers an angle of either 0° (±15°) or 90° (±15°) in eyes with DR was only present in 13.8 % of all cases respectively. Cohen's kappa was used to evaluate the angel categorization of the two graders and there was almost perfect agreement (*k* = 0.889, p < 0.001).

### The FAZ is enlarged in eyes with diabetic retinopathy

**Fig. 3:**
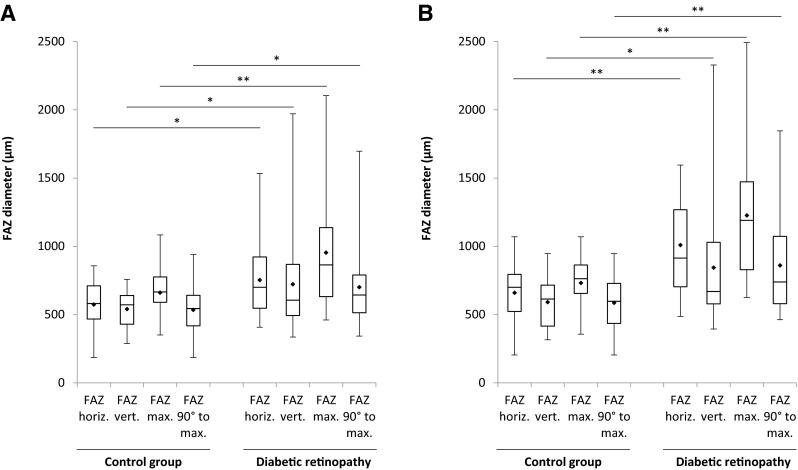
The size of the FAZ diameters within the superficial layer (**a**) and deep layer (**b**). The mean values are indicated by the *dots*. The statistical analyses were performed with the two-sided Mann–Whitney U test (* p < 0.05; ** p < 0.01)

In the superficial layer, the mean horizontal FAZ diameter was significantly larger in the DR group (753 μm ±272 μm) than in the control group (573 μm ±177 μm, *p* = 0.029). As seen in Fig. 3b, the difference of the mean horizontal diameter was even more pronounced in the deep layer with a mean of 659 μm ±194 μm in the control group and 1009 μm ±342 μm in the DR group (*p* = 0.001).

As shown in Fig. 3a and b, the most pronounced difference between the control and DR groups was observed for the maximum FAZ diameter. In the superficial layer, the difference in maximum FAZ diameter between the control group (661 μm ±171 μm) and DR group (953 μm ±393 μm) was pronounced and statistically significant (*p* = 0.008). And in the deep layer, the difference in mean maximum FAZ diameter between the control group (731 μm ±189 μm) and DR group (1227 μm ±484 μm) was even more pronounced (*p* < 0.001).

### Enlargement of the FAZ is correlated with reduced visual acuity

Lastly, the FAZ diameters of all patients were examined for correlation to BCVA.

**Fig. 4:**
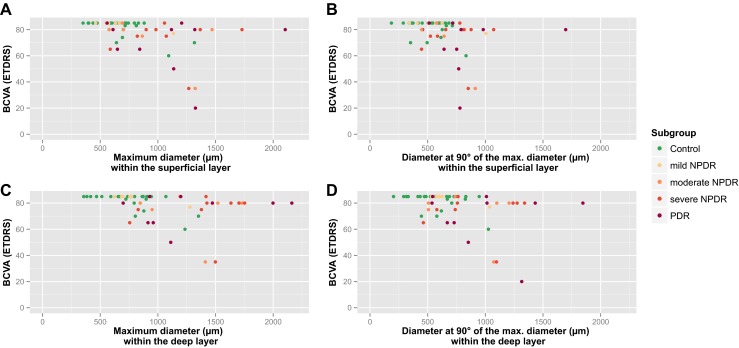
**a** The maximum FAZ diameter in the superficial layer and BCVA (Spearman’s rho = 0.527, *p* < 0.01). **b** The diameter at 90° to the maximum FAZ diameter in the superficial layer and BCVA (Spearman’s rho = 0.401,* p* < 0.01). **c** The maximum FAZ diameter in the deep layer and BCVA (Spearman’s rho = 0.544, *p* < 0.01). **d** The diameter at 90° to the maximum FAZ diameter in the deep layer and BCVA (Spearman’s rho = 0.373, *p* < 0.01)

In the superficial layer, the maximum FAZ diameter and the BCVA (Spearman’s rho = 0.429, *p* < 0.01) as well as the diameter at 90° to the maximum FAZ diameter and the BCVA (Spearman’s rho = 0.347, *p* = 0.01) correlated with the visual acuity, as demonstrated in Fig. 4a and b.

Furthermore, in the deep layer, a correlation of the FAZ diameters with BCVA was also present. Again, the maximum FAZ diameter (Spearman’s rho = 0.490, *p* < 0.01) and the diameter at 90° to the maximum FAZ diameter (Spearman’s rho = 0.306, *p* = 0.025) both correlated with the BCVA.

## Discussion

In this study, we assessed whether OCT angiography can detect changes in FAZ shape. Furthermore, the superficial and deep vasculature surrounding the FAZ was analyzed in DR in comparison to a healthy control group. In addition, we were interested to know if these FAZ alterations correlate with visual acuity and can be used for early screening.

SD-OCT imaging is not able to detect changes in FAZ. Currently, FA is the gold standard for the exact detection of the FAZ, without the possibility to differentiate between superficial and deep retinal vasculature [29].

Our data demonstrate that OCT angiography is one of the first non-invasive imaging techniques capable of detecting changes in the FAZ. OCT angiography data showed that in the superficial layer of the vasculature, the mean horizontal, vertical, maximum and the diameter at 90° to the maximum FAZ diameter were larger in diabetic eyes than in healthy eyes. These findings correspond to previous reports based on funduscopy and FA [3–5]. Moreover, all of the above-mentioned findings are more pronounced in the deep layer, which was unresolvable with previous approaches, especially since the anatomical information of the deep vasculature is lost due to retinal light scattering in FA [14]. Therefore OCT angiography might be a better tool in detecting early FAZ changes in DR compared to FA.

The disintegrity of the vascular arcades not only enlarges the maximum FAZ diameter but also alters the angle of the maximum FAZ diameter [3, 4]. Typically, in healthy eyes, the maximum FAZ diameter was found to be either horizontal (oval shaped FAZ) or vertical (circular shaped FAZ). Angles outside 0° (±15°) or 90° (±15°) were almost exclusively found in diabetic eyes. This observation is of particular importance, as this technique offers an alternative to automated screening. Why capillary loss tends to begin on the above-described angles is unknown.

Ad hoc analysis indicates that in the superficial layer, the calculated sensitivity and specificity of this diagnostic approach would be 93.10 % (95 % confidence interval [CI]: 77.19–98.95 %) and 72.00 % (95 % CI: 50.61–87.88 %), and in the deep layer, 86.21 % (95 % CI: 68.32–96.03 %) and 76.00 % 95 % (CI: 54.87–90.58 %), respectively. Especially in conjunction with the maximum FAZ diameter, the sensitivity and specificity can be further improved.

The FA-based ETDRS grading criteria capillary loss (ischemia) and leakage of fluorescein dye [7] are of particular relevance for clinical practice [6, 30]. Focal photocoagulation, a treatment modality for diabetic macular edema (dME), targets macroaneurysms and microaneurysms with leakage in FA [30]. OCT angiography does not directly depict leakage, and thus does not provide information on blood–retina barrier integrity [26]. However, we found that the overlay of SD-OCT-derived retinal thickness mapping and OCT angiography may help to identify microaneurysms with leakage through local thickness maxima. Hence, OCT angiography might be applicable as a diagnostic tool for planning focal photocoagulation of microaneurysms with leakage [30]. Further research is needed to support this contention.

Correlation of FAZ dimensions with BCVA was found to be statistically significant, which is in line with previous funduscopic and FA reports [3–5]. Nevertheless, FAZ dimensions may not be suitable for predicting BCVA, because of substantial inter-individual variance in dimensions. For instance, one diabetic eye presented with a horizontal FAZ diameter of 1323 μm and a BCVA of 35 ETDRS letters, while another diabetic eye had a BCVA of 80 despite having an horizontal FAZ diameter of 1729 μm. This inter-individual variance might be due to complex differences in foveal development, i.e. growth in axial length after formation of the FAZ [31]. Thus, inter-individual comparison of FAZ size may not be helpful in predicting BCVA, but OCT angiography could be important for longitudinal assessment of FAZ diameter in individual patients and might be of help in detecting early changes without FA [32, 33].

The results of the Diabetes Control and Complications Trial (diabetes type 1) and the United Kingdom Prospective Diabetes Study (diabetes type 2) demonstrated that a 'safe' lower threshold for glycated haemoglobin (HbA1c) does not exist [34, 35]. Thus, OCT angiography of the FAZ might be beneficial for identifying early microvascular abnormalities to determine the appropriate treatment target for patients according to their risk profile (HbA1c < 48 mmol/mol or HbA1c < 53 mmol/mol). Further studies are needed to investigate the utility of OCT angiography in eyes with no or mild DR.

Although the use of the deep vascular layer of OCT angiography seems promising, the automated segmentation for the superficial vascular plexus, deep vascular plexus, outer retina and choriocapillaris was repeatedly imprecise. Especially in patients with diabetic macular edema (dME), which is characterized by fluid accumulation in the outer plexiform layer (Henle fibre layer), the performance of the automated segmentation of the deep vascular layer is suboptimal. The default definition of the borders of the deep layer result in a fixed slab thickness of 55 μm. Thus, in these patients, the outer vasculature of the deep vascular layer is regularly outside the slab. As a result, the examiner must use the manual Z-axis fly-through in these patients.

Furthermore, the instrument requires long fixation on a blue fixation light. Patients with low visual acuity were often especially unable to do so.

### Limitations

The small sample size and the retrospective nature of the study are limitations worth noting. Due to the retrospective nature of the data and the novelty of the technique, the composition of the DR group was heterogeneous (including patients with mild, moderate, severe NPDR and PDR). Because of the retrospective study design, image analysis was not standardized. Further prospective research will be required to confirm our observations.

## Conclusion

OCT angiography is capable of imaging the two major layers of the retinal vasculature without dye injection [26, 27]. Our data show that OCT angiography was able to detect changes in the different retinal layers and in the shape of the FAZ. Since vascular abnormalities are more pronounced in the deep layer, we believe that OCT angiography may be superior to FA for the early detection of DR and for the evaluation of the FAZ in DR. Further studies are needed to evaluate differences in FAZ OCT angiography findings for specified subtypes of DR and other retinal vasculopathies.
